# Exceptional cause of bowel obstruction: rectal endometriosis mimicking carcinoma of Rectum - a case report

**Published:** 2011-11-08

**Authors:** Selim Sassi, Mahdi Bouassida, Hassen Touinsi, Mohamed Mongi Mighri, Sonia Baccari, Fathi Chebbi, Khaled Bouzeidi, Sadok Sassi

**Affiliations:** 1 Department of surgery, Mohamed Thahar Maamouri Hospital, Nabeul, Tunisia; 2 Department of radiology, Mohamed Thahar Maamouri Hospital, Nabeul, Tunisia

**Keywords:** Endometriosis, bowel obstruction, malignancy, surgery, Tunisia

## Abstract

Endometriosis with intestinal serosal involvement is not uncommon in women of childbearing age. However, endometriosis presenting as colon obstruction is rare and occurs in less than 1% of cases. The Lack of pathognomonic signs makes the diagnosis difficult, mostly because the main differential diagnosis is with neoplasm, even during the intervention. Reported here is a case of a 35-year –old woman presenting with bowel obstruction due to rectal endometriosis. The patient presented signs and symptoms of bowel obstruction. Colonoscopy and radiological findings were suggestive of rectal carcinoma. Surgeons performed an anterior resection with right salpingectomy. Histopathology diagnosed bowel endometriosis. This case demonstrates the difficulty of establishing an accurate pre- and intra- operative diagnosis and the ability of intestinal endometriosis to mimic colon cancer.

## Introduction

The most commonly involved areas in women with intestinal endometriosis are the sigmoid colon and rectum. A bowel obstruction complicates recto-sigmoid endometriosis in approximately 10% of cases. Sometimes, making a differential diagnosis of colorectal endometriosis from other malignancies is difficult, due to similar colonoscopy and radiological findings.

We report a case of a 35-year-old woman presenting with acute bowel obstruction. Colonoscopy and radiological findings were suggestive of rectal carcinoma, but the histopathology was not conclusive. The decision was to perform a midline laparotomy, with an anterior resection of the rectum and right salpingectomy. Histopathology of the surgical specimen confirmed that it was endometriosis with hyperplasia involving the full thickness of the bowel wall with no evidence of malignancy.

## Case report

A 35-year woman, 3 gesta 3 para, was admitted for abdominal pain, vomiting and absolute constipation. She had a background of obstructive symptoms, weight loss over a period of 1 year and a history of chronic hematochezia. The patient had regular menses, no history of dyspareunia and these symptoms did not appear during menstruation. Otherwise, her past medical history was unremarkable.

**Figure 1:**
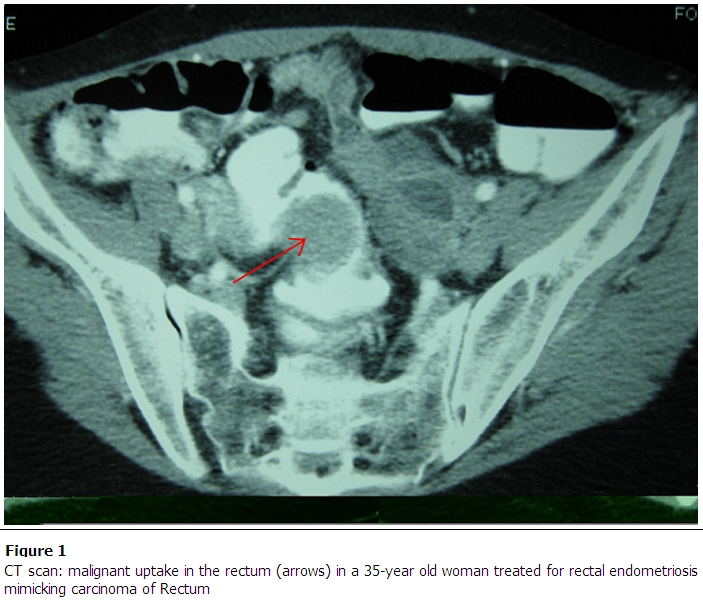
CT scan: malignant uptake in the rectum (arrows) in a 35-year old woman treated for rectal endometriosis mimicking carcinoma of Rectum

Examination and plain abdominal X-ray confirmed the bowel obstruction. Afterwards, a CT scan of the abdomen and the pelvis with IV and rectal contrast was performed, to try to establish the cause of the obstruction, and to help plan the surgical intervention. It suggested a malignant process of the rectum (Figure 1).

The patient underwent elective outpatient colonoscopy that revealed a rectal mass at 8 cm from the rectal sphincters, causing marked stenosis. The colonoscope was unable to pass the lesion but biopsies were taken. Subsequent histopathology was not conclusive showing bleeding tissue.

A lower midline laparotomy was performed, showing a recto-sigmoid mass and a uterus of normal size. The mass had adhesions with the right uterine tube, the posterior lamina of the right broad ligament and the posterior uterine wall. The surgical procedure started with an anterior resection of the rectum and the dissection of the para-colic and intermediate lymph nodes, as well as the ones in the root of the inferior mesenteric artery, ending with an end to end colorectal anastomosis and right salpingectomy.

**Figure 2:**
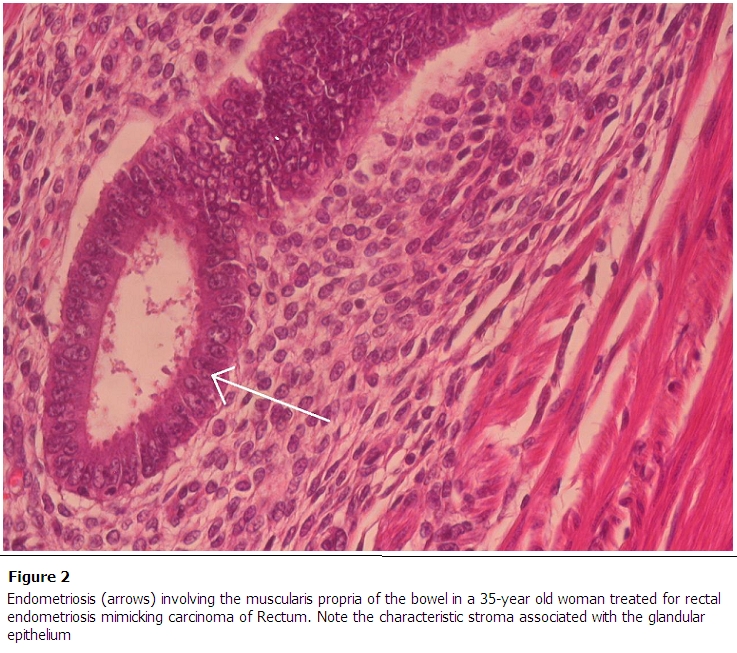
Endometriosis (arrows) involving the muscularis propria of the bowel in a 35-year old woman treated for rectal endometriosis mimicking carcinoma of Rectum. Note the characteristic stroma associated with the glandular epithelium

The specimen was sent to histopathology confirming that it was endometriosis with hyperplasia involving the full thickness of the bowel wall with no evidence of malignancy (Figure 2). It noted also the presence of several benign lymph nodes, one with associated endometriosis.

The postoperative course was uneventful. The patient was discharged after 13 days in good general state. At a follow up after 6 weeks, the patient was well and asymptomatic.

## Discussion

Endometriosis is histologically defined as the presence of endometrial-like tissue outside the uterus. It is reported to affect the intestinal tract in 15%-37% of patients with pelvic endometriosis [1]. This disease is not particularly rare among women of childbearing age [2].

The sigmoid and rectum are involved in 70% of intestinal endometriosis cases, 80% of which are associated to genital endometriosis [3].

Clinical symptoms are present in only one-third of patients with endometriosis of the sigmoid. They are manifested as cramps, flatulence, painful tenesmus, hyper-peristaltis, progressive constipation or diarrhea alternating with constipation. A bowel obstruction complicates sigmoid endometriosis in approximately 10% of cases [4]. A physician can suspect endometriosis, when dealing with a bowel obstruction, if gynecological symptoms are present; such as dyspareunia, infertility or dysmenorrhea. Some patients reportedly display symptoms associated with the menstrual cycle, but these patients represent only about 40% of all patients with endometriosis [2].

Radiological and endoscopic examinations, based on colonoscopy and CT scan, are essential for the diagnosis of intestinal endometriosis. For it may be confused with malignancy, particularly in patients with mucosal involvement. MRI seems to be the most sensitive imaging technique for intestinal endometriosis. However, these methods are not diagnostic [5].

Diagnosis of intestinal endometriosis is difficult. Indeed it can be confused with other more serious lesions such as colon cancer, as demonstrated in this case and in others. But there are a few defining characteristics; for example, endometrial tissue usually involves the outer walls of the colon such as the serosal layer or submucosa. Therefore, a lesion that penetrates the mucosa is less likely to be an endometrial lesion. It is for these reasons that colonoscopy can easily miss a colonic endometrioma.

In conclusion, intestinal endometriosis is a relatively rare disease. And based on clinical symptoms, endoscopic procedure and radiological findings, it can be easily mistaken for malignancy. We suggest thinking of intestinal endometriosis when faced with women of a reproductive age, presenting with gastrointestinal symptoms and an intestinal mass of unknown origin. Some authors suggested Virtual colonoscopy, for it helps to fully describe the lesion ant to visualize the part of the colon proximal to where the endoscope cannot pass. The CT colonoscopy is reported to evaluate the remainder of the colon and to fully characterize the presumed obstructing colon cancer [6].

There is an obvious lack of consensus regarding the treatment of intestinal endometriosis. This is due to the fact that cases are rare presenting with different symptoms but mainly because the diagnosis often comes too late, mostly in mid-surgery. This does not allow an adequate study. In order to define an appropriate treatment, numerous factors must be considered: age, plans for future pregnancies, infertility, pain, as well as intestinal symptoms. The purpose of the treatment for intestinal endometriosis is elimination of symptoms, removal of as much endometrial tissue as possible and cessation of disease progression.

Treatment options consist of medical and surgical treatment. The medications used to treat endometriosis are danazol, high-dose progestins, and GnRH agonists, all of which have equivalent efficiency [5]. Treating with Danatrol or LHRH analogues before surgery may decrease inflammation or vascularisation, thereby facilitating the surgical procedure. A histological diagnosis is needed to begin any treatment. Such combination of medical and surgical treatment has never been formally evaluated [4].

When an obstructive syndrome is present, or in cases of symptomatic advanced endometriosis (defined as reactional fibrosis invading the muscularis of the intestine), the only treatment is surgical resection [7]. This is due to the fact that endometrioic tissue in the bowel muscularis undergoes muscle cell hyperplasia and fibrosis, which make it resistant to medical treatment. We think that Intestinal resection is necessary, along with a salpingo-oophorectomy and hysterectomy in older patients. However, oral contraceptive treatment must be tried in younger patients.

## Conclusion

Endometriosis is a rare cause of intestinal obstruction and it is often mistaken for malignancy. The primary diagnosis is usually of a surgical emergency and there are no specific tests to make a preoperative diagnosis. There is an obvious lack of consensus regarding the treatment of intestinal endometriosis. The treatment is based on surgery and hormonotherapy.
